# The Cuticular Hydrocarbons of *Dasineura Oleae* Show Differences Between Sex, Adult Age and Mating Status

**DOI:** 10.1007/s10886-023-01428-z

**Published:** 2023-04-24

**Authors:** Alice Caselli, Riccardo Favaro, Ruggero Petacchi, Marta Valicenti, Sergio Angeli

**Affiliations:** 1 Center of Plant Sciences, Scuola Superiore Sant’Anna, Piazza Martiri della Libertà 33, Pisa, 56127 Italy; 2 Faculty of Science and Technology, Free University of Bozen-Bolzano, Piazza Università 1, Bolzano, 39100 Italy; 3 Competence Centre for Plant Health, Free University of Bozen-Bolzano, Piazza Università 1, 39100 Bolzano, Italy

**Keywords:** Cecidomyiidae, Cuticular hydrocarbons, GC-MS, Olive leaf gall midge

## Abstract

**Abstract:**

In insects, cuticular lipids prevent water loss and act as semiochemicals. Because of their ecological function, the profile change across the insects’ sex and development offers insight into insect biology and possible tools for pest management. Here, the first work on cecidomyiid cuticular extracts is proposed considering *Dasineura oleae* (Diptera: Cecidomyiidae) males and females at different adult ages (0–12 h, 12–24 h, 24–36 h) and distinct sexual conditions (virgin and mated). A set of 49 compounds were recorded (12 alkanes, 1 monomethyl alkane, 11 fatty acids, 4 esters, 1 aldehyde, 1 allylbenzene, 1 amine, 1 flavonoid, 1 ketone, 1 phenol, 1 steradiene, 1 sterol, 1 terpene, 1 triterpene and 11 unknown compounds), and 18 of them showed significant differences between groups. Among alkanes, hexacosane (*n*C26) exhibited a decreasing trend from the youngest to the oldest females, while pentacosane (*n*C25) and nonacosane (*n*C29) showed a decreasing trend from 0 to 12 h to 12–24 h virgin females. In addition, nonadecane (*n*C19) was significantly more abundant in the youngest males compared to older males and females. The alkanes *n*C25, *n*C26 and *n*C29 have been reported to be age-related also in other dipterans, while *n*C19 has been described as gender-specific chemical cue for platygastrid parasitoids. Further behavioural trials and analyses are required to assign the specific ecological roles to the characterized compounds. Our results may contribute to develop new low-impact control strategies relying on the manipulation of *D. oleae*’s chemical communication (e.g. disruption of mating or species recognition).

**Highlights:**

• Cuticular hydrocarbons are often involved in dipteran intraspecific communication.

• We explored the cuticular profile of *D. oleae* at different age, sex, mating condition.

• Five alkanes and one mono-methyl alkane showed differences among groups.

• Linoleic acid is the most abundant compound in virgins, absent in mated insects.

• Eleven compounds disappear in mated insects, but were present in all virgins.

**Graphical Abstract:**

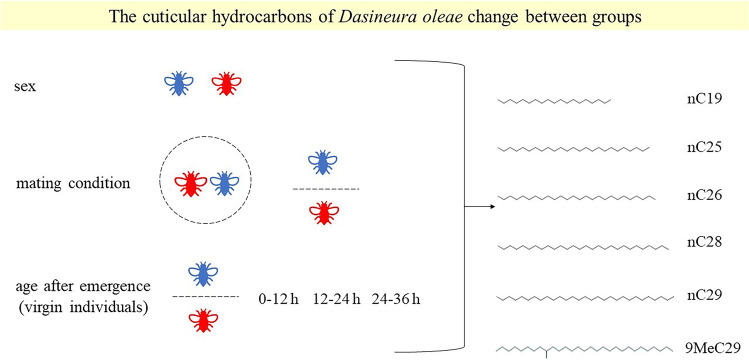

## Introduction

Insect cuticle is covered with a very thin layer of wax consisting of lipids. Among lipids are included hydrocarbons, alcohols, waxes, glycerides, phospholipids, glycolipids, and fatty acids (Drijfhout et al. 2010). Insect cuticular hydrocarbons (CHCs) are essential to withstand environmental stress and desiccation since they cover the entire body surface of insects forming several layers made up of numerous compounds that can be adjusted according to environmental needs (Golebiowski et al. 2011; Chung and Carroll 2015; Otte et al. 2018; Menzel et al. 2019). CHCs are synthetized in secretory cells, called oenocytes, located in the abdominal epidermis (Krupp and Levine 2010) and transferred to insect cuticle (Chung and Carroll 2015). Insect CHCs are classified as long-chain hydrocarbons (20–40 carbons), mainly alkanes, alkenes, alkadienes, methyl-branched alkanes and methyl-branched alkenes (Gibbs 2002; Chung and Carroll 2015; Otte et al. 2018). Their biological functionality is determined by physical properties, such as viscosity and melting point (Menzel et al. 2019). Recently, Menzel et al. (2019) demonstrated that the waterproof characteristic of insect cuticle is due to biphasic CHC layers forming a solid-liquid mixture under ambient conditions. However, even if the main function of CHCs remains desiccation prevention (Mullen et al. 2007), CHCs are also involved in many aspects of chemical communication. Some CHCs play important roles in insect mimicry, labour division (i.e. in social insects) and mating, acting as close-contact pheromones (Ferveur 2005; Howard and Blomquist 2005; Hoffmann et al. 2006; Chung and Carroll 2015; Scolari et al. 2021). Within some insect families, such as Drosophilidae (Jackson and Bartelt 1986), Vespidae (Neves et al. 2012) and Apidae (Vernier et al. 2019), it has been proved that the expression of hydrocarbons undergoes consistent changes in terms of compound composition and concentration during the whole insect development (Butterworth et al. 2020a; Cortot et al. 2022). Particularly, CHCs can be expressed differently in the various developmental stages (Kuo et al. 2012; Butterworth et al. 2019), especially during the pre-reproductive adult phase (Butterworth et al. 2020a), and between the two sexes (Buellesbach et al. 2013; Dapporto et al. 2013; Stinziano et al. 2015). These differences act as indicators of sexual maturity and reproductive viability (Butterworth et al. 2020a). Moreover, in intraspecific relationships, they influence the recognition between species and the attraction of potential mates (Singer 1998; Monnin 2006; Ingleby 2015). Even if the study of CHCs related to insect aging may be an important tool for forensic entomology, few works are nowadays available on this topic (Butterworth et al. 2020a). Furthermore, a better understanding the role of CHCs in insect ecology may lead to the development of new pest control and monitoring (e.g. disruption of mating or species recognition), since for most insect pests the Integrated Pest Management (IPM) and Biological Control strategies rely on manipulating chemical communication (Snellings et al. 2018).

Among dipterans having agricultural importance, the characterization of cuticular compounds has been performed only on few pests, including livestock parasites (e.g. blowflies) (see Braga et al. 2016; Bernhardt et al. 2017; Butterworth et al. 2018, 2020a, b), and fruit flies, such as *Drosophila* spp. (Cobb and Ferveur 1995; Jennings et al. 2014; Snellings et al. 2018; Cortot et al. 2022), *Ceratitis* spp. (Vaníčková et al. 2014), *Bactrocera* spp. (Carlson and Yocom 1986; Galhoum 2017; Park et al. 2020), *Anastrepha* spp. (Vaníčková et al. 2012) and *Zeugodacus cucurbitae* (Carlson and Yocom 1986). To the best of our knowledge, reports on the characterization of cecidomyiid (Diptera: Cecidomyiidae) cuticular profiles are not available so far, even if several gall midges are considered serious crop pests worldwide (Hall et al. 2012).

*Dasineura oleae* (Angelini) (Diptera: Cecidomyiidae) is a gall inducer on *Olea europaea* L., endemic in the Mediterranean Basin (Doğanlar et al. 2011; Simoglou et al. 2012; Tondini and Petacchi 2019; Caselli et al. 2021a; Picchi et al. 2021; Magagnoli et al. 2022). It has been commonly discussed as a minor pest in the olive orchard but starting from 2012 its relevance has increased in many regions of the Mediterranean where several outbreaks have been reported (Picchi et al. 2017; Tondini and Petacchi 2019). To date, notions about the control strategies of this pest are scanty and not up to date. Thus, to be in keeping with IPM control strategies, research on low environmental impact approaches is urgently needed.

Here, we present a descriptive study on *D. oleae* aiming to characterize its CHCs profile. Analyses of virgin and mated individuals of both sexes at different ages were conducted to understand differences among treatments and to lay the foundations for potential alternative control tools against *D. oleae*.

## Materials and Methods

### Insect Collection

Shoots with galled leaves by *D. oleae* were collected during March and April 2021 from an infested olive orchard located in Gavorrano (Grosseto, Italy) (42°54’28.30’’ N, 11°00’10.65’’E) considering the timing of the emergence of *D. oleae* reported by Caselli et al. (2021b) at the same place in 2020. After collection, shoots with a maximum of 2 leaves having 5 galls each were selected and placed in plastic transparent containers (diameter 87 mm, length 115 mm) closed at one extremity with a chiffon fabric (mesh size 0.04 mm) to allow aeration. The shoot stems were immersed in tap water to prevent fast foliar desiccation. Infested olive shoots were replaced every 4 days in order to have fresh specimens.

**Fig. 1:**
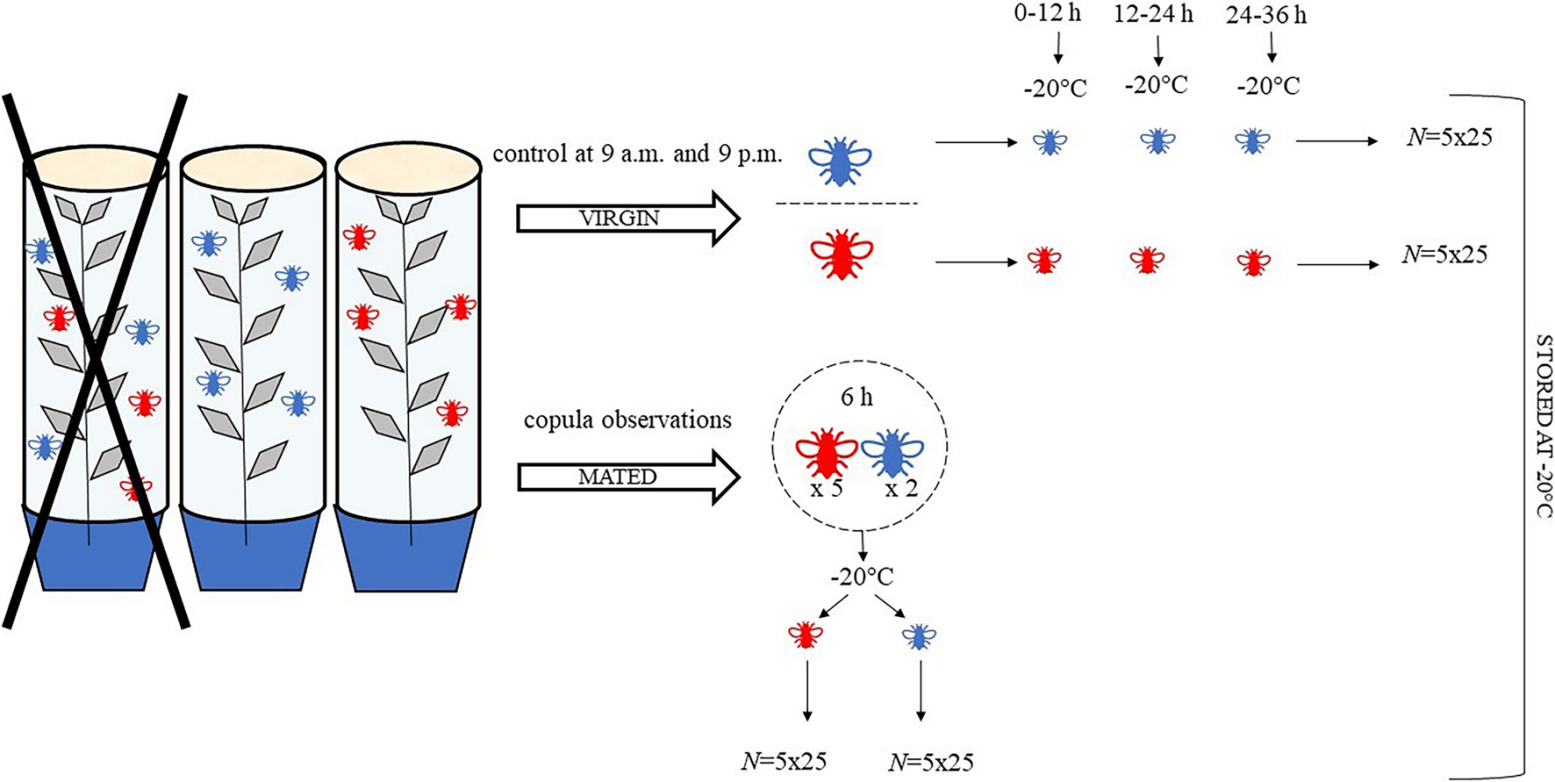
Insect collection.*Dasineura oleae* (Diptera: Cecidomyiidae) was reared to obtain virgin and mated individuals for the extraction of cuticular compounds. Shoots having infested leaves were taken from the field and placed inside transparent containers waiting for adult emergence. Females (red midges) and males (blue midges) found in the same container at the same collection time were discarded. To obtain virgin individuals, containers were controlled every 12 h (9 a.m. and 9 p.m.) and the two sexes were maintained separated. Individuals were frozen (-20 °C) and grouped (*N* = 25) by age (0–12 h, 12–24 h, 24–36 h) for both sexes. Five replicates were done for each age group and samples were frozen at – 20 °C. To obtain mated individuals, 2 virgin males were placed together with 5 virgin females in a Petri dish waiting for the copula. Observations were done to be sure that copulas effectively occurred, and after 6 h (age: 6–18 h) males and females were separated and frozen at -20 °C in groups of 25 individuals. Five replicates per sex were done

From the first emergence, the containers were controlled every 12 h (at 9.00 a.m. and 9.00 p.m.) and the emerged individuals were isolated in single vials (one insect per vial). Insects were maintained alive and starved until they reached a required age (0–12 h, 12–24 h, 24–36 h), then they were killed by freezing (-20 °C) and separated by age in groups of 25 individuals (males and females). For each of the 3 age groups, 5 replicates were done and if both males and females were found in the same container at the same collection time, they were not considered. All groups were stored at -20 °C (Fig. 1).

The collection of mated individuals was based on our laboratory observations, as no mating behaviours (e.g. female calling behavior) have been described for *D. oleae.* We observed that males of this species were capable of mating multiple times, so we placed two virgin males with five virgin females in a Petri dish and observed for copulation to occur. After 6 h, males and females were separated and frozen at -20 °C in groups of 25 individuals (age: 6–18 h). Five replicates per sex were done (Fig. 1).

### CHC Extraction and Analysis

For each sample, the midges (25 individuals of the same age group and sex) were placed in a 1.5 ml GC vial (LGG-Labware, Germany), and 500 µL pentane were added in the vial. The vials were then agitated in an ultrasound bath (ultrasonic cleaner 5510, 40 kHz, Branson, USA) for 2 min. The extract was transferred to a clean vial using a 500 µL glass syringe (Hamilton, Merck KGaA, Darmstadt, Germany), while insects remained in the old vial. The new vials were then opened under a gentle stream of nitrogen gas to allow the solvent to evaporate. Upon complete evaporation, 200 µL of a solution of heptane and pentadecane 10^− 4^, as internal standard, were added to the vial and manually agitated for 1 min to allow solubilization of the dried residues. The samples were analysed by injecting 2 µL into a GC (7890, Agilent Technologies, Santa Clara, USA) splitless mode equipped with a mass selective detector-MS (5975 C, Agilent Technologies). A GC/MS nonpolar HP-5 MS column (Agilent Technology, 30 m x 0.25 mm x 0.25 μm film thickness) was used for separation with a constant helium flow of 1.2 mL/min at an average velocity of 39.62 cm/s. The oven temperature program was started at 35 °C for 2 min and heated to 280 °C at a rate of 7.5 °C/min, held for 5 min. The total run time was 39.66 min. The mass spectrometric detector was operated in scan mode (m/z 20–550 amu). The compounds were initially identified by comparing their mass spectra with those found in two libraries: NIST 20 (National Institute of Standards and Technology 2020) and Wiley 7 (John Wiley, NY, USA); a mixture of n-alkane standards (nC8-nC20, Sigma-Aldrich, St. Louis, Missouri, USA) was used to calculate the linear retention indexes (LRI) of the detected compounds (Van den Dool and Kratz 1963). The compounds were identified based on the comparison of their retention index with the retention indexes reported in the literature (PubChem, Nist, Pherobase). The compounds were finally confirmed by comparing their retention times with those available from synthetic standards (Sigma-Aldrich, St. Louis, Missouri, USA). Compounds that were also detected in a negative control (solvent only) were considered contaminants. The total ion current (TIC) amount of each compound was compared to that of the internal standard pentadecane (*n*C15) 10^− 4^ and the values are reported as a percentage proportion.

### Data Analysis

The statistical analysis was performed using the software R (R Core Team 2021). Each compound was tested for variations due to age (0-12 h, 12-24 h, 24-36 h) and sex (female, male) in virgin individuals and for variations due to mating condition (virgin 12-24 h, mated 6-18 h) and sex (female, male) in virgin and mated individuals by ANOVA test. Values were square root transformed to achieve normalization. A post-hoc pairwise Tukey-test with Bonferroni correction was applied to discriminate among the groups (“multcomp” package, Hothorn et al. 2008).

A dimension reduction principal component analysis (PCA) was performed using the R package “factoextra” (Kassambara and Mundt 2020). The PCA calculated the combination of the compound area data by extracting eigenvalues and eigenvectors of a correlation matrix and then highlighting principal components. A two-dimensional score plot was created to compare the cuticular profiles of the different groups.

All the data are reported as mean ± standard deviation. The figures were created by using the R packages “ggplot2” and “cowplot” (Wickham 2016; Wilke 2020).

## Results

A total of 49 compounds were detected from the analyses of the cuticular extractions of *D. oleae* virgin and mated individuals (Table 1).

**Table 1:** *Dasineura oleae *cuticular compounds

Compound			Females				Males			
	LRI^a^	LRI^b^	Virgin 0–12 h	Virgin 12–24 h	Virgin 24–36 h	Mated 6–18 h	Virgin 0–12 h	Virgin 12–24 h	Virgin 24–36 h	Mated 6–18 h
Aldehydes										
Tetradecanal	1610	1610 (†)	0.53 ± 0.05 (5) a	0.46 ± 0.08 (5) bA	0.4 ± 0.04 (5) c	1.48 ± 0.29 (5) **B**				1.15 ± 0.13 (5) **B**
Alkanes										
Undecane	1100	1100 (†)				0.31 ± 0.12 (3) A				0.14 ± 0.03 (5) A
Pentadecane	1500	1500 (†)	100 ± 0 (5)	100 ± 0 (5)	100 ± 0 (5)	100 ± 0 (5)	100 ± 0 (5)	100 ± 0 (5)	100 ± 0 (5)	100 ± 0 (5)
Hexadecane	1600	1600 (†)								0.15 ± 0.03 (5)
Nonadecane	1900	1900 (†)	0.47 ± 0.05 (4) **a**	0.67 ± 0.12 (5) **a**A	0.66 ± 0.22 (5) **a**	0.48 ± 0.16 (5) B	0.81 ± 0.16 (5) **b**	0.65 ± 0.22 (5) **b**A	0.76 ± 0.13 (5) **b**	0.46 ± 0.12 (5) B
Tricosane	2300	2300 (†)	2.26 ± 2.52 (4) a	2.26 ± 1.27 (3) aA	1.53 ± 1.42 (5) a	0.94 ± 0.6 (5) A			1.01 ± 0 (1) a	0.48 ± 0.43 (5) A
Tetracosane	2400	2400 (†)	0.98 ± 1.06 (5) a	0.72 ± 0.55 (5) aA	0.61 ± 0.35 (5) a	0.62 ± 0.5 (5) A				0.49 ± 0.37 (4) A
Pentacosane	2500	2500 (†)	6.67 ± 3.29 (5) **a**	5.55 ± 2.85 (5) **a**A	8.07 ± 0.98 (5) **a**	4.57 ± 2.91 (5) **A**	2.82 ± 0.15 (5) **a**	2.18 ± 0.34 (5) **a**B	3.39 ± 0.22 (5) **a**	1.85 ± 1.25 (5) **B**
Hexacosane	2600	2600 (†)	3.02 ± 3.25 (5) **a**	2.45 ± 2.39 (5) **b**A	1.56 ± 0.27 (5) **c**	0.83 ± 0.41 (5) **A**	0.65 ± 0.1 (5) **a**	0.68 ± 0.11 (5) **a**	0.71 ± 0.12 (5) **a**	0.4 ± 0.16 (5) **B**
Heptacosane	2700	2700 (†)	10.55 ± 5.79 (5) **a**	8.95 ± 4.79 (5) **a**A	12.89 ± 0.83 (5) **a**	8.76 ± 4.86 (5) A	5.97 ± 0.41 (5) **b**	5.42 ± 0.56 (5) **b**A	6.34 ± 0.39 (5) **b**	4.98 ± 1.42 (5) A
Octacosane	2800	2800 (†)	0.54 ± 0.18 (5) a	0.59 ± 0.18 (5) aA	0.67 ± 0.12 (5) a	0.79 ± 0.33 (5) **A**				0.32 ± 0.33 (5) **B**
Nonacosane	2900	2900 (†)	1.77 ± 0.73 (5) **a**	1.62 ± 0.7 (5) **a**A	2.11 ± 0.22 (5) **a**	1.62 ± 0.57 (5) **A**	1.4 ± 0.31 (5) **b**	1.25 ± 0.17 (5) **b**A	1.16 ± 0.23 (5) **b**	0.78 ± 0.6 (5) **B**
Hentriacontane	3100	3100 (†)	1.98 ± 0.52 (5) a	1.72 ± 0.53 (5) a	2.2 ± 0.56 (5) a		2.75 ± 0.63 (5) a	2.25 ± 0.5 (5) a	2.3 ± 0.5 (5) a	
Allybenzenes										
Eugenol	1355	1355 (†)	0.31 ± 0.01 (5) a	0.35 ± 0.18 (5) a	0.54 ± 0.51 (5) a		0.23 ± 0.07 (5) a	0.19 ± 0.04 (5) a	0.23 ± 0.08 (5) a	
Amines										
N-(2-Phenylethyl) Acetamide	1515	1515 (†)				0.11 ± 0.03 (5) A	0.15 ± 0.05 (5) a	0.1 ± 0.04 (5) a	0.1 ± 0.04 (5) a	0.2 ± 0.15 (5) A
Branched alkanes										
9-Methylnonacosane	2934	2934 (1)	6.18 ± 0.99 (2) a	3.48 ± 2.72 (2) aA	3.46 ± 2.12 (4) a	1.41 ± 0.79 (5) A				2.3 ± 1.4 (5) A
Esters										
Ethyl palmitate	1993	1993 (1)	26.27 ± 50.04 (5) a	7.84 ± 3.34 (5) aA	8.58 ± 4.28 (5) a	0.53 ± 0.3 (5) B	8.3 ± 4.59 (5) a	9.21 ± 2.81 (5) aA	8.04 ± 3.54 (5) a	0.3 ± 0.15 (5) B
Methyl linoleate	2092	2092 (1)	5.27 ± 1.71 (5) a	12.64 ± 6.74 (5) bA	15.65 ± 10.07 (5) c	1.48 ± 0.91 (5) B	10.12 ± 4.48 (5) a	16.36 ± 1.97 (5) bA	12.52 ± 5.36 (5) c	0.99 ± 0.93 (5) B
Ethyl linoleate	2161	2161 (†)	22.74 ± 5.87 (3) b	129.19 ± 90.45 (5) a	80.68 ± 13.99 (5) a		58.25 ± 38.84 (4) a	76.84 ± 7.55 (4) a	70.92 ± 12.22 (5) a	
2-Linoleoylglycerol	2418	2418 (2)	1.57 ± 0.16 (5) a	0.98 ± 0.4 (5) aA	0.65 ± 0.12 (2) a	5.73 ± 2.10 (2) **B**	1.58 ± 0.54 (5) a	1.26 ± 0.57 (5) aA	0.88 ± 0.48 (5) a	0.36 ± 0.11 (4) **B**
Fatty acids										
Caprylic acid	1174	1174 (†)	0.37 ± 0.08 (5) a	0.32 ± 0.04 (4) aA	0.34 ± 0.02 (3) a	0.29 ± 0.09 (5) A	0.3 ± 0 (1) a	0.34 ± 0.05 (2) aA	0.36 ± 0.04 (2) a	0.16 ± 0.04 (5) **B**
Pelargonic acid	1270	1270 (†)	0.64 ± 0.08 (5) a	0.48 ± 0.23 (5) aA	0.56 ± 0.24 (5) a	0.61 ± 0.18 (5) A	0.36 ± 0.04 (5) a	0.36 ± 0.03 (5) aA	0.49 ± 0.13 (5) a	0.37 ± 0.14 (5) A
Capric acid	1366	1366 (†)	0.94 ± 0.05 (5) **a**	0.63 ± 0.07 (5) **b**B	0.43 ± 0.08 (5) **c**	0.39 ± 0.21 (4) A	0.72 ± 0.08 (5) **a**	0.57 ± 0.08 (5) **b**B	0.49 ± 0.07 (5) **c**	0.25 ± 0.16 (5) A
Lauric acid	1562	1562 (†)	4.45 ± 0.54 (5) **a**	2.85 ± 0.29 (5) **b**A	1.53 ± 0.12 (5) **c**	1.09 ± 0.51 (5) B	3.2 ± 0.42 (5) **a**	2.48 ± 0.33 (5) **b**A	1.94 ± 0.36 (5) **c**	0.88 ± 0.44 (5) B
Myristoleic acid	1745	1755 (3)	0.8 ± 0.17 (5) **a**	0.59 ± 0.09 (5) **b**	0.35 ± 0.09 (4) **c**		0.62 ± 0.12 (5) **a**	0.51 ± 0.1 (5) **b**	0.28 ± 0.01 (5) **c**	
Myristic acid	1758	1758 (1)	5.78 ± 0.15 (5) **a**	3.8 ± 0.5 (5) **b**A	2.32 ± 0.16 (5) **c**	0.78 ± 0.65 (5) B	4.43 ± 0.92 (5) **a**	3.13 ± 0.67 (5) **b**A	1.77 ± 0.17 (5) **c**	0.66 ± 0.42 (5) B
Palmitoleic acid	1939	1939 (1)	3.27 ± 1.67 (5) a	2.4 ± 0.44 (5) bA	1.25 ± 0.36 (5) c	0.78 ± 0.57 (4) B	3.06 ± 0.85 (5) a	1.96 ± 0.65 (5) bA	1.14 ± 0.3 (5) c	0.57 ± 0.37 (5) B
Palmitic acid	1967	1967 (1)	83.17 ± 44 0.13 (5) a	65.04 ± 6.98 (5) bA	35.8 ± 3.86 (5) c	40.46 ± 26 (5) B	73.1 ± 10.74 (5) a	43.65 ± 9.09 (5) bA	29.09 ± 1.84 (5) c	31.84 ± 20 (5) B
Oleic acid	2098	2098 (†)	5.61 ± 4.47 (5) a	9.51 ± 5.1 (5) a	6.05 ± 1.79 (5) a		5.46 ± 1.99 (5) a	6.84 ± 1.52 (5) a	5.83 ± 0.87 (5) a	
Linoleic acid	2148	2148 (†)	268.3 ± 153.56 (5) a	220.57 ± 120.89 (5) a	208.35 ± 15.39 (5) a		307.97 ± 77.41 (5) a	237.03 ± 82.04 (5) a	188.41 ± 19.17 (5) a	
Stearic acid	2171	2172 (1)	44.18 ± 6.04 (5) a	37.2 ± 1.9 (3) a			36.65 ± 7.65 (5) a			
Flavonoids										
7,9-Di-tert-butyl-1-oxaspiro(4,5)deca-6,9-diene-2,8-dione	1914	1916 (1)	0.44 ± 0.18 (3) a	0.39 ± 0.05 (5) a	0.46 ± 0.11 (5) a		0.4 ± 0.06 (5) a	0.44 ± 0.08 (5) a	0.51 ± 0.07 (5) a	
Ketones										
β-Ionone	1355	1355 (†)	1.1 ± 0.09 (5) **a**	1.04 ± 0.19 (5) **a**A	0.97 ± 0.14 (5) **a**	0.2 ± 0.13 (2) B	0.61 ± 0.12 (5) **a**	0.49 ± 0.05 (5) **a**A	0.57 ± 0.14 (5) **a**	0.08 ± 0.01 (3) B
Phenols										
α-Tocopherol	3132	3130 (1)	5.67 ± 2.41 (5) **a**	5.5 ± 0.74 (5) **a**	4.85 ± 2.49 (5) **a**		10.98 ± 1.14 (5) **a**	9.73 ± 1.05 (5) **a**	9.22 ± 1.24 (5) **a**	
Steradiens										
3,5-Stigmastadiene ^*^	3084		2.15 ± 0.95 (5) a	1.88 ± 0.83 (5) aA	1.88 ± 0.3 (5) a	0.27 ± 0.09 (4) B	2.77 ± 0.47 (5) a	2.3 ± 0.38 (5) aA	1.87 ± 0.5 (5) a	0.44 ± 0.17 (5) B
Sterols										
γ-Sitosterol ^*^	3302	3290 (1)	3.46 ± 1.91 (5) a	4.54 ± 0.67 (4) aA	4.84 ± 0.98 (5) a	1.49 ± 1.16 (4) A	5.17 ± 0.73 (5) a	5.49 ± 1.33 (5) aA	4.83 ± 0.61 (5) a	1.62 ± 0.24 (2) A
Terpenes										
Dihydroactinidiolide	1522	1522 (2)	0.76 ± 0.05 (5) **a**	0.65 ± 0.1 (5) **a**	0.65 ± 0.1 (5) **a**		0.39 ± 0.04 (5) **a**	0.36 ± 0.05 (5) **a**	0.42 ± 0.1 (5) **a**	
Triterpenes										
Squalene	2828	2828 (†)	0.37 ± 0.07 (3) a	0.54 ± 0.15 (3) aA	0.61 ± 0.18 (5) a	0.97 ± 0.5 (4) A	0.6 ± 0.41 (3) a	0.44 ± 0 (1) aA	0.56 ± 0.25 (5) a	0.61 ± 0.42 (5) A
Unknowns										
Unknown 1	1085		0.4 ± 0.06 (5) a			0.49 ± 0.09 (5) A				0.31 ± 0.03 (5) A
Unknown 2	1121		0.49 ± 0.03 (5) a	0.36 ± 0.02 (5) a	0.34 ± 0.06 (3) a		0.36 ± 0.05 (4) a	0.3 ± 0.02 (4) a	0.37 ± 0.05 (5) a	
Unknown 3	1176									0.06 ± 0 (5)
Unknown 4	1224					0.27 ± 0.08 (5) A				0.19 ± 0.01 (5) A
Unknown 5	1277		0.31 ± 0.01 (4)							
Unknown 6	2249		16.8 ± 31.38 (5) a	35.84 ± 55.61 (3) a			4.07 ± 3.33 (4) a	2.96 ± 2.29 (3) a		
Unknown 7	2665		0.5 ± 0.13 (5) a	0.38 ± 0.05 (5) a	0.37 ± 0 (1) a		0.47 ± 0.02 (5) a	0.43 ± 0.09 (5) a	0.36 ± 0.14 (3) a	
Unknown 8	2733		3.41 ± 5.92 (5) a	2.84 ± 5.04 (5) aA	0.57 ± 0.07 (5) a	0.49 ± 0.27 (5) A	0.94 ± 0.13 (5) a	0.72 ± 0.13 (5) aA	0.98 ± 0.04 (5) a	0.56 ± 0.37 (5) A
Unknown 9	2944		4.02 ± 1.79 (3) **a**	5.23 ± 0.11 (3) **aA**	3.84 ± 0.82 (3) **a**	0.82 ± 0.49 (5) **B**	8.95 ± 0.73 (5) **b**	8.3 ± 0.81 (5) **bA**	8.79 ± 0.84 (5) **b**	1.67 ± 0.93 (5) **B**
Unknown 10	3219		1.83 ± 0.78 (5) a	1.68 ± 1.48 (5) aA	1.6 ± 1.15 (5) a	1.02 ± 0.89 (5) A	1.67 ± 0.58 (5) a	1.54 ± 0.73 (5) aA	1.07 ± 0.56 (5) a	2.14 ± 1.23 (5) A
Unknown 11	3330		2.54 ± 1.04 (5) a	2 ± 0.68 (5) aA	1.77 ± 0.75 (5) a	0.82 ± 0.63 (5) A	2.78 ± 0.96 (5) a	2.38 ± 0.71 (5) aA	2.28 ± 0.27 (5) a	1.74 ± 0.86 (5) A

Summary table of the cuticular compounds identified by GC/MS analysis of the solvent extraction of *D. oleae* females and males, mated (age: 6–18 h) and virgin at three different timings after emergence (age: 0–12 h, 12–24 h, 24–36 h) and their amounts (relative area % ± SD in comparison to the internal standard C15). Each sample was collected from 25 insects by rinsing with pentane. The compounds were identified by mass spectrometry and confirmed by comparison of linear retention indexes from the literature or retention times from synthetic standards when available. The number of samples where the compound was found is reported between brackets. Compounds in grey shading show statistically significant changes between conditions. Lowercase letters show difference between ages of virgin midges, whilst uppercase letters report difference between mating condition (12-24 h virgins vs. 6-18 h mated). The bold font indicates differences between sex

LRI^a^ = Linear retention index calculated in relation to n-alkanes

LRI^b^ = Linear retention index already published in peer-reviewed journals and listed in Pubchem (1), NIST Webbook (2) or Pherobase (3). When possible, the LRI was verified by a synthetic standard (†).

*= Putatively annotated compound

Anova test was used to analyse the effect of sex and age in virgins and the effect of sex and mating condition between 12-24 h virgins and 6-18 h mated. Comparisons between groups were performed with post-hoc Tukey tests

Authors’ observations allow to affirm that each male mated more than once with different virgin females, while females appeared to be monogamous.

**Fig. 2:**
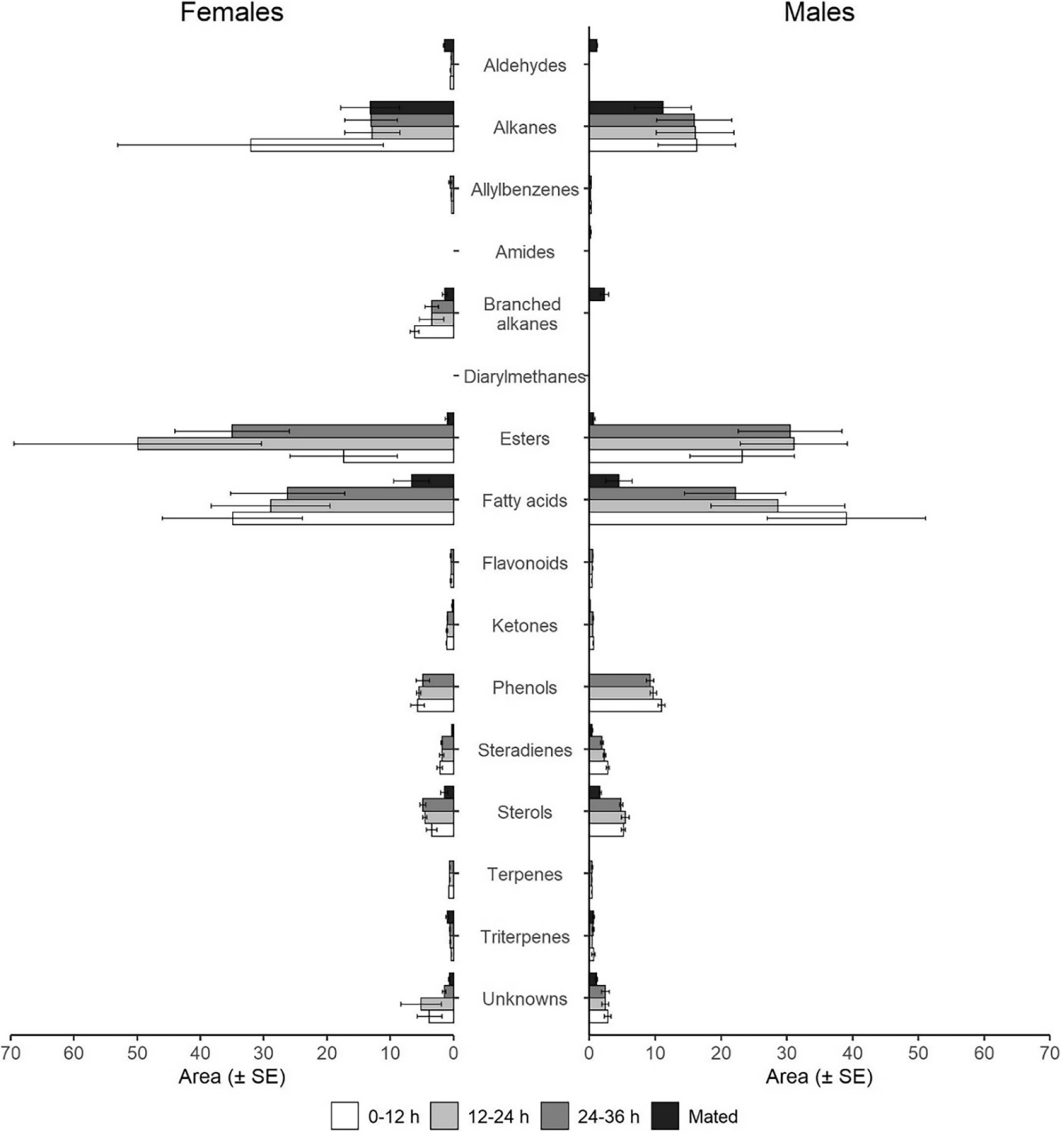
Classes of chemical compounds collected in cuticular extractions of female and male *Dasineura oleae *(Diptera: Cecidomyiidae). Virgin individuals at different age (1 = 0–12 h, 2 = 12–24, 3 = 24–36), and mated individuals at one age (6–18 h) were tested

Identification was not possible for 11 compounds as reported in the last part of Table 1. To better understand differences among chemical classes, compounds showing statistically differences were grouped as reported in Fig. 2.

### Compounds of Virgins

Among the collected compounds, 13 were characteristic of only virgin males and females, and only one was present in females of 1st age (i.e. unknown 5) and 2 were present in individuals of 1st and 2nd age (i.e. stearic acid and unknown 6). Ethyl linoleate was one of the compounds absent in mated individuals, and its amount remained constant within virgin males, while it significantly increased in 12–24 h females (*F*_2,24_=7.45, *p* = 0.0038). Myristoleic acid showed a decreasing amount with age in both sexes (*F*_2,24_=36.40, *p* < 0.001) and an overall lower amount in males (*F*_1,24_=10.95, *p* = 0.031). The amount of α-tocopherol, absent in mated individuals, did not follow any trends, but in males was almost double to that in females (*F*_1,24_=42.76, *p* < 0.001). Dihydroactinidiolide was lower in virgin males than in virgin females (*F*_1,24_=99.9, *p* < 0.001).

### Compounds of the Mated

Four compounds found only in mated individuals; two of them where characteristic of both sexes (i.e. undecane, unknown 4), and two were found only in mated males (i.e. unknown 3, hexadecane). Other compounds were instead absent from the virgins of a sex and appeared only after mating. Tetradecanal was present only in virgin females and decreased with age (*F*_2,24_=91.24, *p* < 0.001), but in mated individuals of both sexes it was at least two-fold higher (*F*_1,12_=100.91, *p* < 0.001), even if mated males had less than mated females (*F*_1,12_=7.17, *p* = 0.021). Like tetradecanal, octacosane was not present in virgin males, appearing then in mated ones in a lower amount than in mated females (*F*_1,12_=7.78, *p* = 0.016). The amine N-(2-phenylethyl) acetamide was detected in all the males but in the females only after mating. The amount in mated females and virgin males averages about half than in the mated males but no significant difference was found between the groups (*F*_2,12_= 1.89, *p* = 0.26).

### Compounds that Decrease in Mated

The amount of ethyl hexadecanoate was significantly lower in mated females and males (*F*_1,16_=135.9, *p* < 0.001) whilst linoleoylglycerol increased in females after mating but decreased in males (*F*_1,12_=71.06, *p* < 0.001). The amount of 3,5-stigmastadiene was significantly lower in mated individuals of both sexes (*F*_1,15_=81.84, *p* < 0.001) compared to the virgins. Caprylic acid decreased in mated males in respect to mated females (*F*_1,12_=8.74, *p* = 0.012) and virgin males (*F*_1,10_=6.58, *p* = 0.024), but not in mated females (*F*_1,12_=1.03, *p* = 0.16). β-Ionone was lower in virgin males (*F*_1,24_=98.2, *p* < 0.001) and decreased significantly after the mating (*F*_1,11_=121.8, *p* < 0.001). The amount of nonadecane (*F*_1,16_=0.09, *p* = 0.76), methyl linoleate (*F*_1,16_=0.33, *p* = 0.57) did not show any differences between mated females and males. Nonadecane was more abundant in virgin males (*F*_1,23_=4.81, *p* = 0.038), but decreased after mating (*F*_1,16_=7.13, *p* = 0.016). Also methyl linoleate decreased in mated insects (*F*_1,16_=93.20, *p* < 0.001).

### Compounds that Decrease with Age

Capric acid differed between sex of virgins (*F*_1,24_=4.45, *p* = 0.045) and decreased with age (*F*_1,24_=53.88, *p* < 0.001) and mating condition (*F*_1,15_=17.53, *p* < 0.001). Similarly, lauric acid differed between sex of virgins (*F*_1,24_=6.04, *p* = 0.021) and decreased with age (*F*_1,24_=91.14, *p* < 0.001) and mating condition (*F*_1,16_=59.35, *p* < 0.001). Palmitic acid decreased with age (*F*_2,24_=8.24, *p* = 0.002) and after mating (*F*_1,16_=5.47, *p* = 0.032). Palmitoleic acid amount decreased with age (*F*_2,24_=11.33, *p* = 0.0003) and in mated (*F*_1,15_=35.40, *p* < 0.001). Myristic acid (*F*_2,24_=94.17, *p* < 0.001) showed the same trend, decreasing with age in both virgin sexes, and was lower in males (*F*_1,24_=21.87, *p* < 0.001). Furthermore, the amount of myristic acid did not differ between the sex of mated midges (*F*_1,16_=1.06, *p* = 0.33).

### Compounds that Differ Between Sex

Dihydroactinidiolide was lower in virgin males than in virgin females (*F*_1,24_=99.9, *p* < 0.001). Heptacosane was lower in virgin males (*F*_1,24_=7.07, *p* = 0.013). Unknown 9 was higher in males than in females both virgin (*F*_1,24_=98.81, *p* < 0.001) and mated (*F*_1,14_=29.32, *p* < 0.001) but decreased after mating (*F*_1,14_=157.4, *p* < 0.001). A decreasing trend was recorded for hexacosane in virgin females (*F*_2,24_=3.25, *p* = 0.024) and its amount was overall lower in males, both virgin (*F*_1,24_=16.13, *p* = 0.0005) and mated (*F*_1,16_=8.32, *p* = 0.0108). 9-Methylnonacosane was not recorded in virgin males but appeared in the mated ones. The amount of pentacosane was lower in males than in females, both in virgin (*F*_1,24_=22.13, *p* < 0.001) and mated (*F*_1,16_=7.88, *p* = 0.012). The same was reported for nonacosane in virgin (*F*_1,24_=7.82, *p* = 0.01) and mated males (*F*_1,16_=5.61, *p* = 0.03).

Having used pentadecane as an internal standard did not allow to assess whether this compound is part of the insect cuticular hydrocarbons. However, statistical analysis did not reveal differences between pentadecane absolute values (*F*_7,32_= 1, *p* = 0.49), which were instead extremely similar, with no variation between groups.

**Fig. 3:**
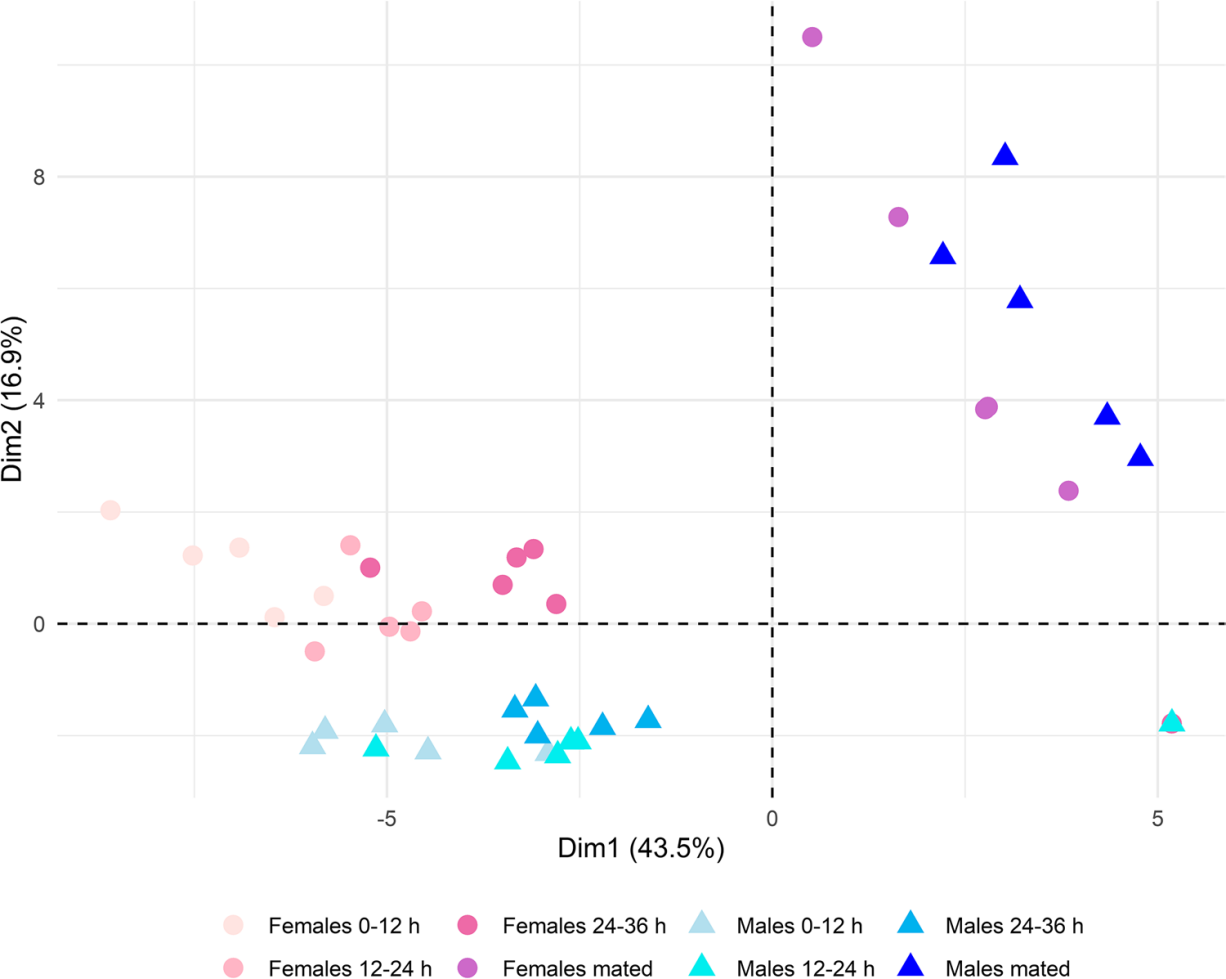
Principal component analysis score plot of the cuticular extracts obtained from pools of individualsof *Dasineura oleae* (Diptera: Cecidomyiidae). Pools are reported according to sex (males and females) and mating condition (mated and virgin). Virgin individuals were analysed at different times from emergence (0–12 h, 12–24 h, 24–36 h), while mated individuals had one age (6–18 h)

PCA allowed to determine the clustering of the cuticular compounds based on sex and age (Fig. 3). The individuals are clearly clustered together, showing a general transition along PC1 (43.5%) according to age. While the virgins are divided by sex on PC2 (16.9%), the transition to the mated individuals showed a remarkable deviation along the principal components.

## Discussion

Dipteran cuticular hydrocarbons have received wide attention, but no information is currently available on any cecidomyiid species. Here, the first report on cuticular extracts characterizing a gall midge, *D. oleae*, is proposed.

Insect cuticular hydrocarbons are mainly present in two forms, as saturated (alkanes) and unsaturated compounds (alkenes) (Sharma et al. 2020). In this work, five linear alkanes and one monomethyl alkane showed differences between groups, while no alkenes were recorded. Among alkanes, pentacosane, octacosane and nonacosane were present. These compounds are three main components of the *Aedes aegypti* L. (Diptera: Culicidae) cuticular profile and are used in medical entomology to estimate female mosquito age (Desena et al. 1999; Gerade et al. 2004). Similarly, in the *D. oleae* cuticular profile, the female amount of pentacosane and nonacosane changes with age. Pentacosane is also known as a fertility signal in the subsocial bee *Ceratina calcarata* Robertson (Hymenoptera: Apidae), acting as a precursor of queen sexual pheromone (Pizzi and Rehan 2021). It is particularly abundant in sexually mature flies belonging to *Chrysomya varipes* (Macquart) (Diptera: Calliphoridae) species (Butterworth et al. 2020a), and it is a cuticular component of the *Drosophila melanogaster* Meigen (Diptera: Drosophilidae) profile (Rajpurohit et al. 2016). Contrary to pentacosane and nonacosane, the amount of octacosane remained unvaried among the ages of virgin females but changed after mating. Octacosane seems to be a common insect hydrocarbon, since it is also present in the cuticular profile of *Blattella germanica* (L.) (Blattodea: Blattellidae) (Augustnowicz et al. 1986), *Melipona quadrifasciata* (le Peletier) (Hymenoptera: Apidae) (Borges et al. 2012) and workers of *Oecophylla smaragdina* (Fabricus) (Hymenoptera: Formicidae) (Azhagu Raj et al. 2017). Hexacosane showed a decreasing trend from the youngest to oldest virgin females of *D. oleae*, as already demonstrated for the mosquito *Ae. aegypti* (Polerstock et al. 2002). It is also present in the female cuticular profile of other dipterans, such as *Lucilia cuprina* (Wiedemann) and *Hemilucilia segmentaria* (Fabricus) (Diptera: Calliphoridae) (Rocha Barbosa et al. 2017). In our study, the amount of nonadecane was almost double in 0–12 h virgin males compared to 0–12 h virgin females. Differences of this compound among sexes have been already documented in the stink bug *Nezara viridula* (L.) (Hemiptera: Pentatomidae), where its egg parasitoid, *Trissolcus basalis* (Wollaston) (Hymenoptera: Platygastridae), uses nonadecane as a gender-specific chemical cue of *N. viridula*, showing a significant preference for females which extract did not contain this compound (Colazza et al. 2007). Since several species of *D. oleae* parasitoids are platygastrids (Stavraki 1970; Moallem 1975; Doğanlar 2011; Baidaq et al. 2015; Batta 2019; Tondini and Petacchi 2019), the implication of nonacosane in *D. oleae*-parasitoid interactions requires further investigation. The only methyl branched alkane recorded in our study was 9-methylnonacosane. It is known to be part of the epicuticular profile of the redbanded stink bug *Piezodorus guildinii* (Westwood) (Heteroptera: Pentatomidae), an important pest of soybean crops in South America (Sessa et al. 2021).

Environmental factors (e.g. air, water vapor, ultraviolet radiation) can alter and breakdown insects’ cuticular hydrocarbons disrupting chemical communication (Hatano et al. 2020). Therefore, some species have adapted to this phenomenon by emitting volatile organic compounds (VOCs) that might be recorded in cuticular extracts (Hatano et al. 2020). For example, the volatile long-range pheromones often included aldehydes originated through the oxidation (e.g. photooxidation, autoxidation) of non-volatile cuticular waxes (Bartelt et al. 2002). Moreover, whole body extraction may also allow to dissolve non-cuticular compounds as for instance those present in the secretion of exocrine glands (Provost et al. 2008). In our study, tetradecanal was typical of *D. oleae* virgin females and mated individuals of both sexes. It is known as the most abundant aldehyde from males of the sawfly *Cephus cintus* Norton (Hymenoptera: Cephide) (Bartelt et al. 2002), and as one of the representative components of the *Acheta domesticus* (L.) (Orthoptera: Gryllidae) profile (Warthen and Uebel 1980). Furthermore, tetradecanal was found to have different roles depending on sex, as in the case of *Rossomyrmex minuchae* Tinaut (Hymenoptera: Formicidae), in which tetradecanal has been recorded as the major component of queen Dufour’s glands having a repellent effect on ants belonging to another species that is usurped by *Rossomyrmex* queens for new host nests (Ruano et al. 2005). In *Bombus* spp. Latreille (Hymenoptera: Apidae) tetradecanal is instead used by males as a territorial-marking pheromone (Urbanová et al. 2004). Being tetradecanal found in mated males (but in lower amount than in mated females), we question whether it could be passed from the females to the males during the mating, and what possible ecological role it might have. The same was observed also for octacosane, thus suspecting a similar route of transmission while mating.

Among fatty acids found in the cuticular extracts of *D. oleae*, myristic acid, myristoleic acid and palmitoleic acid significantly decreased in virgin individuals from the youngest to oldest. Concerning the mating condition, it is interesting to note that linoleic acid is present just in virgin individuals and completely absent in mated ones, and its amount in all the virgin ages is 2–3 times more abundant compared the internal standard. However, to the best of our knowledge a potential role of these acids in insect chemical communication is still uninvestigated. Fatty acids methyl and ethyl esters are common in the honeybee (*Apis mellifera* L.), and are fundamental for the chemical communication of larvae, workers and queen (Slessor et al. 2005). Furthermore, in the solitary bee *Osmia rufa* (Hymenoptera: Megachilidae) they constitute the main components of the sexual pheromone (Krieger et al. 2006). In our work, ethyl linoleate and methyl linoleate were recorded having different amounts between groups, but no literature was found on their ecological role as pheromones in insects. Furthermore, we recorded hexadecenoic acid ethyl ester and linoleoylglycerol, showing differences in mated individuals. Ethyl hexadecanoate is known as a volatile compound of *Protaetia brevitarsis* Lewis (Coleoptera: Scarabeidae) larvae (Yeo et al. 2013), while in adult insects it is a gender specific compound of *Bicyclus martius sanaos* (Hewitson) (Lepidoptera: Nymphalidae) males (Wang et al. 2014). Ethyl hexadecanoate has been previously identified in *Heliconius* butterflies as a component of a complex mixture of esters, acids, and hydrocarbons acting as anti-aphrodisiac transferred to males from females during mating (Wang et al. 2014). In our study however, this compound did not show any gender specificity and the lowest amount was found in mated females, inducing to consider another function compared to *Heliconius* butterflies. Linoleoylglycerol has been found as a component of fatty larval tissue of *D. melanogaster* (Tortoriello et al. 2013). However, no other information on its role as insect compound is currently available.

In this study, α-tocopherol was absent in mated individuals and showed its major abundance in young males and females. Generally, it is not considered as a compound for chemical communication, but is commonly found in insects’ food sources, as for *D. melanogaster* (Parker and McCormick 2005). Even if the feeding habits of *D. oleae* adults are currently unknown, we speculate that α-tocopherol may be extracted from the cuticula of the gall midge as a larvae food contaminant. Indeed, α-tocopherol is normally found in olive leaves and its amount depends on *O. europaea* variety (Tarchoune et al. 2019). α-Tocopherol has been found also in the nutritional analysis results of *Hermetia illucens* L. larvae, *Chilecomadia moorei* Silva larvae and *Musca domestica* L. adults, used as feed species for captive insectivorous (Finke 2012). Another probable food contaminant we found in the *D. oleae* profile was 3,5-stigmastadiene, a basic compound in oils and fats (Piironen et al. 2000). 3,5-Stigmastadiene is commonly used as marker to ascertain the authenticity of vegetable oils (Gallina Toschi et al. 1996; Giuffrè 2021), such as olive (Crews et al. 2014), argan (Mohammed et al. 2021), avocado (Flores et al. 2019) and grape seed (Matthäus 2008), since high amounts of this compound have been associated with exposure to high temperatures and refining, normal practices in food frauds (Piironen et al. 2000; Flores et al. 2019). In the *D. oleae* cuticular profile, mated individuals of both sexes showed a significantly lower amount of 3,5-stigmastadiene, suggesting an energy consumption due to copula or a partial loss following the time after emergence. A similar consideration can be made for γ-sitosterol, as it is a common compound of plant leaves, including those of *O. europaea* (Gül and Şeker 2006; Canbarro et al. 2019).

In our study, the amine N-(2-phenylethyl)acetamide was found in males and mated females. It has been described as a secondary component of the trail pheromonal blend released by the rectal bladders of *Camponotus* spp. (Hymenoptera: Formicidae) (Kohl et al. 2001, 2003). Furthermore, N-(2-phenylethyl)acetamide has been detected in the anal secretions of *Nicrophorus vespilloides* Herbst (Coleoptera: Silphidae) as a probable regulator of carrion microbial colonization (Degenkolb et al. 2011). To the best of our knowledge, this compound has been frequently isolated as a biologically inactive by-product of antibiotic fermentation (Degenkolb et al. 2011), but it has never been associated to insect sex or mating condition. Its presence in only the mated females may represent a sign of the successful mating, being therefore passed from the male to the female as a marking pheromone. This hypothesis could explain why N-(2-phenylethyl)acetamide was not present in the virgin females, and fits with the observed mating behaviour, where each male mates with several virgin partners. Mated females were observed to be ignored by males (authors’ personal observation). Such compounds have been reported as a mate-guarding strategy to prevent other males’ courtship in various insect species (Laturney and Billeter 2016). In *Drosophila melanogaster*, (*Z*)-vaccenyl acetate and (*Z*)-7-tricosene, have been associated with chemical mate-guarding. These are produced respectively in the ejaculatory bulb and in oenocytes cells in the subepidermal abdomen and cause reduced male courtship and mating delays when applied on the females or released in the environment (Zawistowski and Richmond 1986; Ejima et al. 2007). Whether such an effect can be elicited in *D. oleae* requires further investigations and behavioural trials.

## Conclusions

The study of the *D. oleae* cuticular profile showed a clear set of 49 compounds. Among them cuticular hydrocarbons such as alkanes and one monomethyl alkane were recorded. Furthermore, other compounds (e.g. fatty acids) were registered. Eighteen compounds were found to exhibit variations in relation to either mating condition or age after emergence. We suggest that these differences, having effect on courtship and mating, may be considered for developing potential alternative control methods, as already suggested for other dipteran pests (e.g. *Drosophila suzukii*). Anyway, to gain a deeper understanding of their potential role in chemical communication of *D. oleae*, additional studies utilizing behavioural trials are necessary.

## Data Availability

Not applicable.
